# Supplementary material for: Immunoproteasome functions explained by divergence in cleavage specificity and regulation
Source: eLife. 2017 Nov 28;6:e27364. doi: 10.7554/eLife.27364 (PMC5705213; doi:10.7554/eLife.27364)
Supplement: Supplementary File 1. — Percent identity was compared across replicates for the intact library (‘no enzyme control’) and after the addition of the immunoproteasome as an example. The 8 hr time point was chosen to maximize the number of cleavages in the comparison. [file elife-27364-supp1.docx]

| **Technical Replicate** | **Technical Replicate** | **% Identity** |
| --- | --- | --- |
| No Enzyme Control #1 | No Enzyme Control #2 | 79.5 |
| No Enzyme Control #1 | No Enzyme Control #3 | 76.7 |
| No Enzyme Control #2 | No Enzyme Control #3 | 79.4 |
| immunoproteasome 8h #1 | immuoproteasome 8h #2 | 70.4 |
| immunoproteasome 8h #1 | immuoproteasome 8h #3 | 68.1 |
| immunoproteasome 8h #2 | immuoproteasome 8h #3 | 71.4 |
