# Supplementary material for: Immunoproteasome functions explained by divergence in cleavage specificity and regulation
Source: eLife. 2017 Nov 28;6:e27364. doi: 10.7554/eLife.27364 (PMC5705213; doi:10.7554/eLife.27364)
Supplement: Supplementary File 2 [file elife-27364-supp2.docx]

| **Substrate** | ***K*_m_**  **(μM)** | ***k*_cat_**  **(s^-1^)** | ***k*_cat_/*K*_m_**  **(μM^-1^s^-1^)** | **Ratio *k*_cat_/*K*_m_ (i20S/c20S)** | **Ratio *V*_max_ (i20S/c20S)** |
| --- | --- | --- | --- | --- | --- |
| **EWHW-*ACC* (parent)** |  |  |  |  |  |
| **i20S** | 52 | 6.8 | 0.13 | 8.4 | 6.0 |
| **c20S** | 73 | 1.1 | 0.015 |  |  |
| **EWFW-*ACC* (optimized)** |  |  |  |  |  |
| **i20S** | 7.0 | 4.8 | 0.69 | 4.0 | 16 |
| **c20S** | 1.8 | 0.31 | 0.17 |  |  |
| **Ac-ANW-*AMC* (commercial)** |  |  |  |  |  |
| **i20S** | 20 | 1.2 | 0.058 | 6.0 | 12 |
| **c20S** | 9.8 | 0.10 | 0.010 |  |  |
| **Iso-VQA-*ACC ^a^* (optimized)** |  |  |  |  |  |
| **i20S** | 43 | 0.65 | 0.015 | 0.33 | 0.28 |
| **c20S** | 51 | 2.3 | 0.045 |  |  |

*^a^* Michaelis-Menten parameters could not be readily determined for the corresponding commercial substrate (Ac-WLA-*AMC*) due to >80-fold reduced specific activity (pmol/s/μg) at 10 μM substrate concentration.
