# Supplementary material for: Immunoproteasome functions explained by divergence in cleavage specificity and regulation
Source: eLife. 2017 Nov 28;6:e27364. doi: 10.7554/eLife.27364 (PMC5705213; doi:10.7554/eLife.27364)
Supplement: Supplementary File 3. [file elife-27364-supp3.docx]

| **Detection Method** | **Subunit** | **CFZ** | **ONX 0914** | **PR-825** |
| --- | --- | --- | --- | --- |
| **EWFW-*ACC*** | LMP7 | 81 | 60 | 510 |
| **Iso-VQA-*ACC*** | β5 | 33 | 524 | 31 |
| **LLVY-*AMC*** | LMP7 / β5 | 53 | 256 | 216 |
| **ProCISE** | LMP7 | 79 | 43 | 656 |
| **ProCISE** | β5 | 27 | 579 | 37 |
